# Navigating real-world challenges: A case study on federated learning in computational pathology

**DOI:** 10.1016/j.jpi.2025.100464

**Published:** 2025-07-23

**Authors:** Lydia A. Schoenpflug, Ruben Bagan Benavides, Marta Nowak, Fahime Sheikhzadeh, Arash Moayyedi, Kamil Wasag, Jacob Reimers, Michael Zhou, Raghavan Venugopal, TumorProfiler Consortium, Bettina Sobottka, Yasmin Koeller, Michael Rivers, Holger Moch, Yao Nie, Viktor H. Koelzer

**Affiliations:** a Department of Pathology and Molecular Pathology, University Hospital and University of Zurich, Zurich, Switzerland; b Roche Diagnostics, Digital Pathology, Santa Clara, CA, United States; c Roche Diagnostics (Schweiz) AG, Rotkreuz, Zug, Switzerland; d Institute of Medical Genetics and Pathology, University Hospital Basel, Basel, Switzerland; e Department of Biomedical Engineering, University of Basel, Basel, Switzerland

**Keywords:** Computational pathology, Federated learning, Digital immune phenotyping, Distributed systems, Healthcare infrastructure

## Abstract

Federated learning (FL) allows institutions to collaboratively train deep learning models while maintaining data privacy, a critical aspect in fields like computational pathology (CPATH). However, existing studies focus on performance improvement in simulated environments and overlook practical aspects of FL. In this study, we address this need by transparently sharing the challenges encountered in the real-world application of FL for a clinical CPATH use case. We set up a FL framework consisting of three clients and a central server to jointly train deep learning models for digital immune phenotyping in metastatic melanoma, utilizing the NVIDIA Federated Learning Application Runtime Environment (NVIDIA FLARE) across four separate networks from institutes in four countries. Our findings reveal several key challenges: First, the FL model performs the best across all clients' test sets but does not outperform all local models on their own client test set. Second, long experiment duration due to system and data heterogeneity limited experiment frequency, alleviated by optimizing local client epochs. Third, infrastructure design was hindered by hospital and corporate network restrictions, necessitating an open port for the server, which we resolved by deploying the server on an Amazon Web Services infrastructure within a semi-public network. Lastly, effective experiment management required IT expertise and strong familiarity with NVIDIA FLARE to enable orchestration, code management, parameter configuration, and logging.

Our findings provide a practical perspective on implementing FL for CPATH, advocating for greater transparency in future research and the development of best practices and guidelines for implementing FL in real-world healthcare settings.

## Introduction

Artificial intelligence (AI)-driven computational pathology (CPATH) algorithms are powerful tools in analyzing gigapixel whole slide images (WSIs).^1^ Simultaneously, one of the most significant barriers to AI adoption in pathology is the limited access to large, high-quality, labeled imaging datasets. One paradigm addressing this challenge is federated learning (FL). FL enables multiple institutions to collaboratively develop an AI model utilizing their own local data, without the need for data sharing, thereby distributing the burdens of data availability, annotation, and computation across participants, while ensuring data privacy.

Although whereas implementation strategies for non-medical, real-world FL settings exist, these often focus on “cross-device” models (e.g., mobile device FL) which differ significantly from the “cross-silo” approach required for CPATH.^2^, ^3^, ^4^ The cross-device model involves thousands of clients and small data packets, whereas the cross-silo CPATH model is defined by a small number of institutional partners, large datasets, and significant computational hardware at each site. Therefore, this study aims to investigate the practical challenges arising from these CPATH-specific constraints: namely, navigating stringent institutional network security, managing large, gigapixel-scale data, and hardware heterogeneity, as well as overcoming logistical hurdles posed by a limited number of international clients.

The application of FL to pathology is still in an early stage of adoption, with few studies conducting experiments in a non-simulated, multi-institutional setup.^2^ Here, we employ a FL approach to the immune phenotyping analysis of cancer samples from the Swiss TumorProfiler Observational Clinical Trial (TuPro)^5^ as well as two independent population-based cohorts in a real-world clinical setting, making it the first study investigating FL for a segmentation and cell detection tasks in immunohistochemistry (IHC) images. We identify four areas in which we encountered challenges in setting up and running real-world FL experiments for a CPATH use case and provide unique insights into addressing these challenges: 1) Performance: Optimally training a DL model in a federated setting not only requires consideration of classical DL algorithm design, such as model architecture and training paradigms, but also FL-specific methodologies such as aggregation algorithms. This has been the primary focus of previously published real-world FL studies for both CPATH^6^^,^^7^ and other healthcare applications.^8^, ^9^, ^10^, ^11^, ^12^, ^13^, ^14^, ^15^, ^16^, ^17^, ^18^, ^19^ 2) Experiment duration: Depending on the task and factors such as system and data heterogeneity, federated training can take significantly longer than centralized training. Addressing and optimizing run time therefore is a crucial component of FL algorithm development. 3) Infrastructure design: The hardware and network requirements, including the optimal placement of the FL server and clients, are not specified in prior publications,^6^^,^^7^ we therefore transparently share our setup and the identified requirements. 4) Experiment management: Orchestrating, configuring, and logging FL experiments is essential for efficient and reproducible research. We share our experiment management experience with the NVIDIA FLARE software framework,^20^ developed by NVIDIA Corporation (Santa Clara, CA, USA).

## Methods

This section details the FL experimental and infrastructure design of this study. We train digital immune phenotyping (DIP) algorithms, consisting of a tumor segmentation and a CD8+ cell detection model in a federated manner. Further details on the datasets, task of DIP, as well as the model training of the DIP algorithms can be found in Supplementary Materials S1–S3.

### Federated learning experimental design

**Fig. 1:**
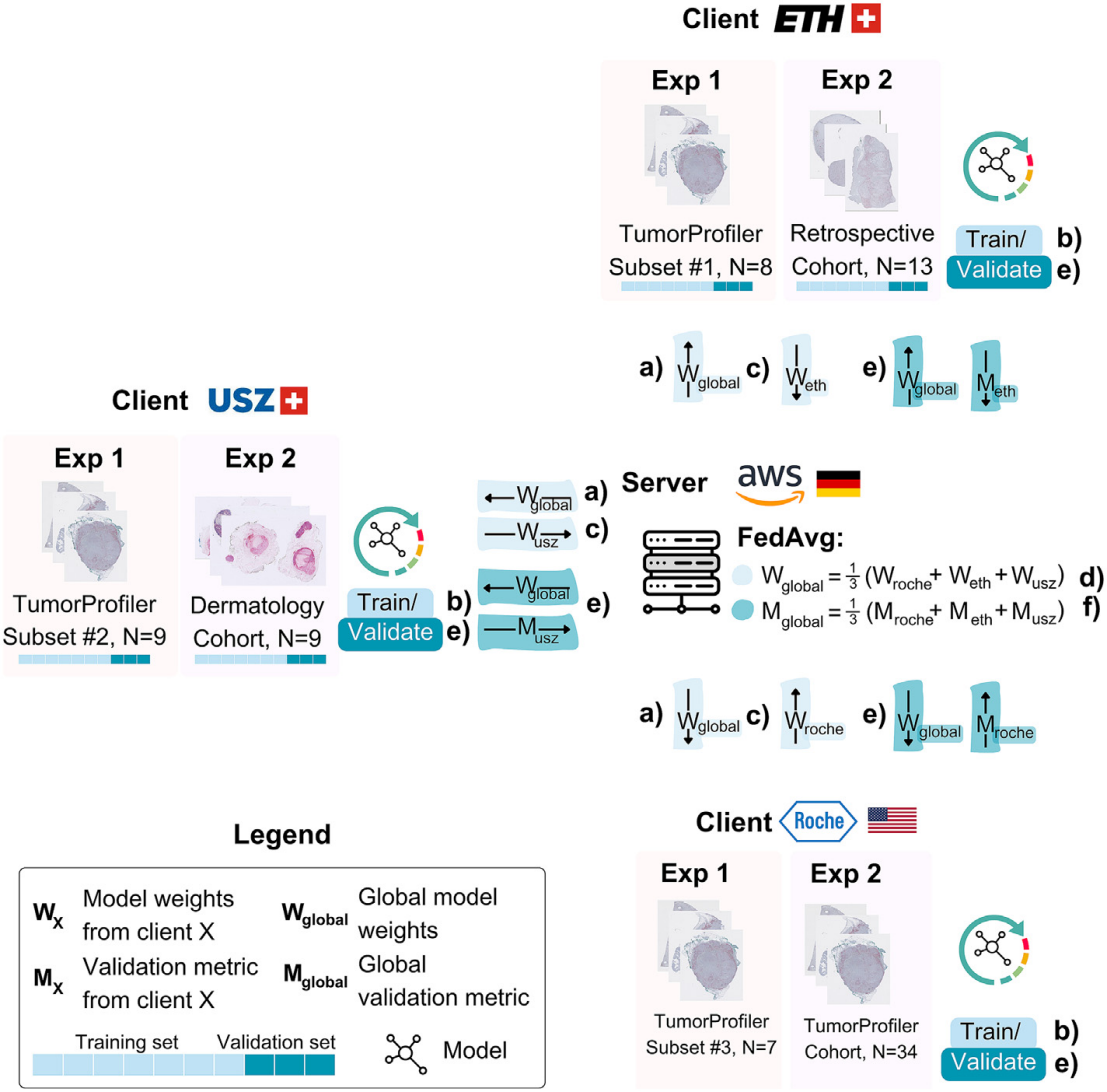
FL experiment workflow. First, we start with client-side global model initialization,where the model is initially pre-trained on the TuPro cohort before the first FL round begins. Then, we conduct multiple FL Rounds (*N* < maximum number of rounds allowed), a round consists of steps (a)–(e), which are highlighted in the figure: a) The server sends the current global model to all clients, (b) local model training on each client (Supplementary Material S3), (c) the clients send back the locally trained models to the server, (d) aggregation of the local models into an updated global model using FedAvg^21^ on the server, (e) evaluation of the global model on the clients validation set to, (f) check if the convergence condition is met. When the global model converges, or all FL rounds are completed, the final, best-performing global model is evaluated on the clients' held-out test sets.

We utilized the open-source, domain-agnostic NVIDIA FLARE framework v.2.3.9^20^ to set up a FL client-server infrastructure and integrated our DIP algorithms into the framework. We conducted the following experiments (Table 1), each following the workflow displayed in Fig. 1:

1. **Single-cohort, proof of concept:** We trained the DIP models on the TuPro Cohort in a centralized and federated manner. For the federated training, the TuPro training set was split into three client training sets on a case-level stratified label split, representing an independently and identically distributed (IID) data setting. Federated and centralized model performance were evaluated on the TuPro held-out test set.
2. **Multi-cohort, real-world setting:** We trained a FL model on three independent cohorts (TuPro, Retrospective, and Dermatology Cohort) which were not shared across the clients and have varying label distributions and WSI appearance, representing a non-IID data setting. FL model performance was evaluated on each client's held-out test set and compared to each client model, which was trained through centralized learning on each client's local cohort, and therefore referred to as CL-tupro, CL-retro, CL-derma, respectively. The comparison to the FL model performance reflects the institution's benefit in joining the FL framework, compared to developing a model based on their available data only. The following settings were investigated: a) Baseline, b) with stain augmentation, and c) varying the number of client-side local training epochs during each FL round to investigate the effects on experiment duration.

**Table 1:** Experimental design: Dataset distribution and hyperparameter settings for the conducted experiments. (a): Experiment 1: Single-cohort, proof of concept; (b): Experiment 2: Multi-cohort, real-world setting. CV: cross-validation, N/A: not applicable.

(a) Experiment 1: Single-cohort, proof of concept				
		Federated		
		FL clients		
	Centralized	Roche	USZ	LeoMed
Training dataset	TuPro Training (*n* = 24)	TuPro Training Subset #1 (*n* = 7)	TuPro Training Subset #2 (*n* = 9)	TuPro Training Subset #3 (*n* = 8)
Validation dataset	TuPro Validation (*n* = 10)			
Test dataset	TuPro Test (*n* = 80)			
Local FL epochs	N/A	20 epochs		
Max. epochs/ rounds	400 epochs	150 FL rounds		
Early stopping	Criterion: F1 score Patience: 20 epochs	Criterion: F1 score Patience: 20 FL rounds		


(b) Experiment 2: Multi-cohort, real-world setting						
	Centralized			Federated		
	CL baselines from clients			FL clients		
	CL-TuPro	CL-Derma	CL-Retro	Roche	USZ	LeoMed
Train + validation dataset	TuPro 3-fold CV (*n* = 34)	Derma 3-fold CV (*n* = 9)	Retro 3-fold CV (*n* = 13)	TuPro 3-fold CV (*n* = 34)	Derma 3-fold CV (*n* = 9)	Retro 3-fold CV (*n* = 13)
Test dataset	TuPro Test (*n* = 80)	Derma Test (*n* = 13)	Retro Test (*n* = 20)	TuPro Test (*n* = 80)	Derma Test (*n* = 13)	Retro Test (*n* = 20)
Local FL epochs	N/A			**Experiment 2A** **+** **B**: 20 epochs **Experiment 2C**: 1, 5, 20 epochs		
Max epochs/rounds	150 epochs			150 FL rounds for local epochs = 20 1000 FL rounds for local epochs = 1, 5		
Early stopping	Criterion: F1 score Patience: 20 epochs			Criterion: F1 score Patience: 20 FL rounds

### Federated learning infrastructure

Our client-server FL setup consists of three clients and a central server, distributed across four separate networks from institutes in four countries (Table 2). Each participating institution was required to operate a client capable of communicating with the server and providing the necessary GPU and CPU hardware infrastructure to support the FL task and host the datasets. This setup involved placing the server in a semi-public network accessible only to whitelisted client IPs, while ensuring that the clients also whitelisted the server IP. It is important to note that the available hardware resources varied significantly among the clients, depending on each institution's capacity, resulting in a heterogeneous setup.

**Table 2:** Location, hardware, and dataset specification for all clients in the FL infrastructure. E1: Experiment 1, E2: Experiment 2.

	Institution	Data	Location	Network setup	Hardware type	GPU	CPU	RAM
Server	Amazon Web Services (AWS)	No	Frankfurt, Germany	Roche semi-public network, Open port Whitelisted Client IPs	T2 2XLarge	No	8 Cores (Intel Xeon E5–2686 v4)	32 GB
Roche client	Roche Diagnostics	E1: TuPro #1 E2: TuPro	Santa Clara, US	Roche Internal Network, Whitelisted Server IP	DGX	NVIDIA A100 (40GB)	64 Cores (AMD EPYC 7742)	1 TB
USZ client	University Hospital Zürich (USZ)	E1: TuPro #2 E2: Derma	Zürich, Switzerland	USZ research network, Whitelisted Server IP	GPU Workstation	NVIDIA TITAN V (12 GB)	16 Cores (Intel Core i7-7820×)	64 GB
LeoMed client	ETH Zürich	E1: TuPro #3 E2: Retro	Zürich, Switzerland	ETH semi-public network Whitelisted Server IP	Tenant in LeoMed, a HPC for sensitive data	NVIDIA GeForce RTX 3090 (24 GB)	15 Cores (AMD EPYC-Rome Processor)	57 GB

## Results and practical challenges

### Challenge #1: Performance

In the single-cohort, proof-of-concept Experiment 1, the FL models perform as well as (±1%) or better (>1%) than the centralized models in terms of mean F1 score for tumor segmentation and mean average precision (mAP) for CD8+ cell detection (Table 3a). This demonstrates that FL is a feasible approach for our tasks.

**Table 3:** Performance results for the FL experiments. For tumor segmentation, the mean is computed as the F1 score across all classes, for CD8+ cell detection the mAP represents the area under the precision-recall curve. (a) Experiment 1: Results on the TuPro test set, comparing a centralized setup where all TuPro training data are stored in a single location, to a federated setting with the data distributed in an IID manner across three clients. (b) Experiment 2C: Experiment duration and model performance on the client held-out test sets for various number of client-side local epochs. Mean F1 and mAP range from 0 to 1 with higher values indicating better performance.

(a)		
	Tumor segmentation, Mean F1	CD8+ cell detection, mAP
Centralized	**0.816**	0.675
FL	0.806	**0.704**


(b)										
	Tumor segmentation, Mean F1					CD8+ cell detection, mAP				
Local epochs	Derma cohort	Retro cohort	TuPro Cohort	Mean	Run time	Derma cohort	Retro cohort	TuPro Cohort	Mean	Run time
1	0.49	0.56	0.60	0.55	**4d**	0.63	0.72	0.66	0.67	**0.71d**
5	0.61	0.68	0.73	0.67	10.3d	**0.66**	0.75	0.71	**0.71**	2.8d
20	**0.69**	**0.75**	**0.80**	**0.75**	25.2d	**0.66**	**0.76**	**0.72**	**0.71**	5.7d

**Fig. 2:**
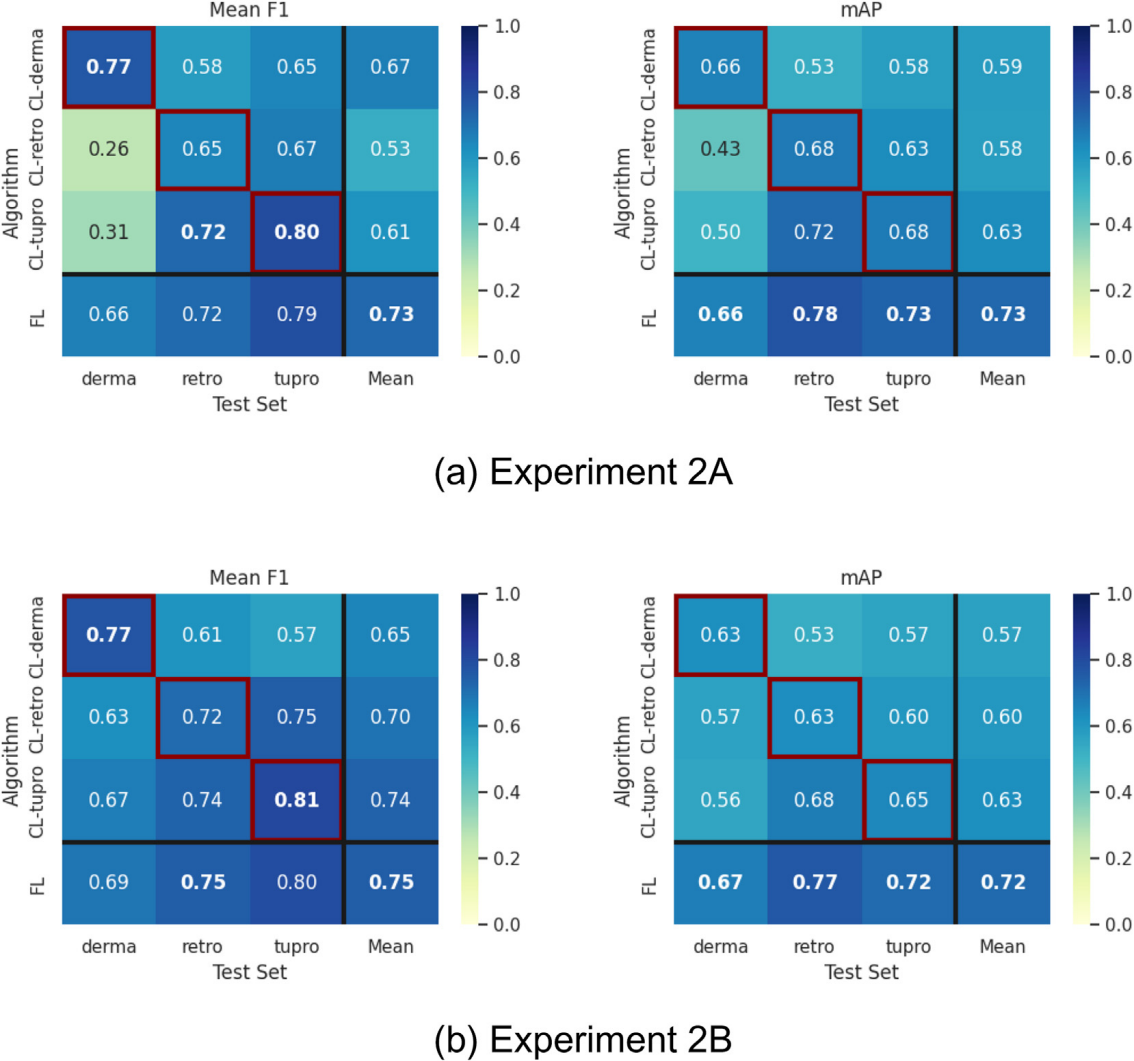
Performance results for Experiment 2A and 2B. For tumor segmentation, the mean is computed as the F1 score across all classes, for CD8+ cell detection the mAP represents the area under the precision-recall curve. Left column: Tumor segmentation performance in terms of mean F1 score; Right column: CD8+ cell detection performance in terms of mAP, (a) Experiment 2A: Baseline, (b) Experiment 2B: With stain augmentation.

In multi-cohort Experiment 2A, the FL model for CD8+ cell detection shows performance equivalent to or better than each client model across all test sets (Fig. 2a). For tumor segmentation, several observations are made: 1) The FL model demonstrates the best generalization performance across all clients' test sets. 2) The CL-derma model outperforms the FL model on the Dermatology cohort by a mean F1 score of 0.11. 3) The CL-retro and CL-tupro models perform significantly worse on the Dermatology test set, achieving a mean F1 score less than 0.35, whereas CL-derma achieves a mean F1 greater than 0.5 on the Retrospective and TuPro test sets.

In Experiment 2B, we examined the impact of stain augmentation,^22^ keeping all other hyperparameters consistent. Its introduction results in slightly decreased CD8+ cell detection mAP (−0.02) for the CL-derma and FL models and equivalent performance with a lower mAP variance across cohorts for CL-tupro and CL-retro (Fig. 2b). Regarding tumor segmentation, we note an increase in overall mean F1 for CL-retro (+0.17), CL-tupro (+0.13), and the FL model (+0.02) (Fig. 2). Only the CL-derma model does not benefit from stain augmentation, suggesting that the inherent high variation in WSI appearance within the Dermatology cohort already facilitates generalization to staining variation.

### Challenge #2: Experiment duration

In Experiment 2C, we investigated the impact of varying the numbers of local, client-side training epochs. This was motivated by the long duration of Experiments 2A and 2B and aimed to determine the optimal balance between performance and time to convergence. For CD8+ cell detection, we found that halting training after 5 local epochs yielded performance comparable to that of 20 epochs, effectively halving the training time. However, for tumor segmentation, this was not the case; training for 20 local epochs resulted in an 8% higher mean F1 score compared to just 5 epochs.

### Challenge #3: Infrastructure design

**Fig. 3:**
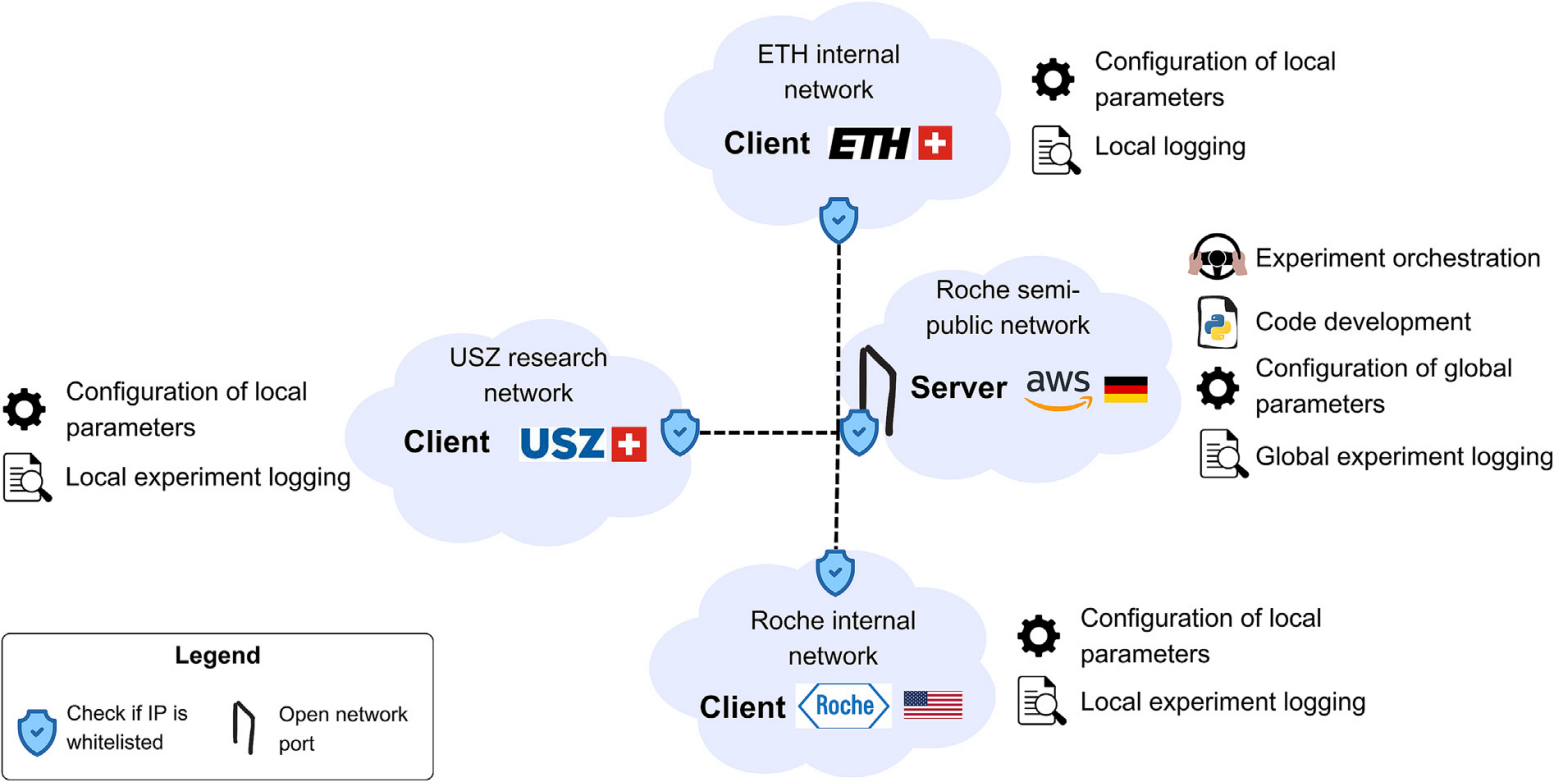
FL Infrastructure and experiment management design.

The main challenge in setting up our FL infrastructure was establishing a secure connection between clients and the server. To address this, the server needed to open a port, which posed a security risk in corporate and hospital networks. Consequently, the FL server was hosted on an AWS server within a Roche semi-public network (Fig. 3), access is restricted to only whitelisted IPs. Additionally, the USZ client was placed within a research network to enable communication with the server, as inbound and outbound traffic in the standard hospital network is restricted.

### Challenge #4: Experiment management

Our experiment management setup involved several sub-tasks and challenges (Fig. 3). First, it required both local, client-side, and global, server-side configuration. For the server-side, we used NVIDIA FLARE's configuration files to set hyperparameters and define the experiment workflow, including training, aggregation, and validation steps. Client-side configuration was implemented based on Hydra.^23^ Second, we optimized experiment orchestration, by custom-implementing early stopping and checkpointing to save and load the latest and best-performing global model and make our code publicly available at https://github.com/NVIDIA/NVFlare/pull/3506. Third, the code for federated training of our DL models was managed via a server-side GitLab repository, enabling collaborative development, whereas NVIDIA FLARE ensured distribution of the code package to all clients at the start of the experiment. Lastly, we implemented a logging tool, where all the logs and metrics generated by the clients are collected by the server and uploaded as a package to our GitLab repository.

## Discussion

FL is an essential technology for multi-institutional CPATH algorithm development. To our knowledge, this study is the first to offer detailed insights into the practical challenges encountered in applying FL to a clinical CPATH use case, particularly for IHC-stained image analysis. The following challenges we encountered are not merely technical hurdles but directly caused by “cross-silo” CPATH-specific constraints, fundamentally distinguishing it from the “cross-device” models common in mobile applications.

First, we focus on the challenges concerning the performance of our FL DIP model. Consistent with previous research on FL in CPATH,^6^^,^^7^^,^^24^, ^25^, ^26^, ^27^ the FL DIP models achieve performance comparable to a centralized model when trained on identical datasets. However, in the multi-cohort context, the FL model, despite strong cross-cohort generalizability, does not necessarily surpass the performance of locally trained models at each client site. This is a classic challenge in cross-silo FL, where a small number of institutional partners contribute highly heterogeneous, complex datasets. In contrast to mobile FL, where data heterogeneity stems from diverse user behaviors and environments, FL for CPATH needs to address the complexity introduced by different institutional protocols and data acquisition methods. This leads to our attempt to employ stain augmentation as a method for data alignment. Our findings suggest a key lesson learned: for CPATH, simple data alignment via stain augmentation is insufficient, warranting more generalizable image representation techniques, such as foundation-model-based feature extraction.^28^ However, whereas foundation models have been extensively explored for H&E image analysis, their generalization to IHC images has not been thoroughly validated,^29^ requiring further study under a centralized learning paradigm before studied under a FL paradigm.

Second, a significant challenge and limiting factor was the duration of experiments a direct consequence of the CPATH model's massive data and hardware requirements. Some experiments required several days to complete because of the gigapixel-size images and system heterogeneity across clients. This long duration, constrained by the slowest client who may have the least powerful hardware resources and/or largest dataset, severely reduces experimental frequency. Whereas mobile FL focuses on mitigating communication bottlenecks from thousands of quick updates, the core challenge in CPATH FL is the baseline run time aiming for model convergence in each round. Given fixed client datasets and hardware, adjusting local epochs became our primary tool for balancing performance and time, a critical consideration for any practical CPATH FL implementation.

The third challenge involves the design of the FL infrastructure, which is perhaps the most defining differentiator of the cross-silo FL paradigm. The initial phase of identifying suitable hardware and determining network placement took several weeks. Unlike mobile clients on open networks, our clients exist within secure hospital and corporate environments. This requires placing the server in a semi-public network with an open port and carefully whitelisting IPs for each client. This logistical and security hurdle is a primary, foundational challenge for real-world medical FL that is largely absent from other FL paradigms. We therefore recommend closely collaborating with IT network experts from each institution before setting up the infrastructure to ensure efficient and functional deployment of clients and the server.

Lastly, we highlight challenges associated with experiment management. The complexity and high stakes of long-running CPATH experiments necessitate flexible and sophisticated frameworks like NVIDIA FLARE.^20^ The most significant and time-consuming aspect was implementing custom components to extend NVIDIA FLARE, such as a user-friendly configuration system and early stopping mechanism, a level of granular control required when each client is a significant research partner and each experimental run is a major investment of time and resources.

Previous research has primarily focused on methodological advancement^6^^,^^7^ to improve performance, guiding users in selecting aggregation algorithms, and mitigating data heterogeneity.^30^, ^31^, ^32^ However, other crucial aspects necessary for effective FL research, such as ensuring efficient experiment durations, designing FL infrastructure, and managing experiments, remain unaddressed. This oversight is problematic, as these factors significantly impact the feasibility, efficiency, and success of FL experiments. Wang et al.^33^ underscores this gap with regards to FL for radiology, and is the first to provide insights into practical, healthcare-focused FL design. We expand on this by presenting a CPATH use case and highlighting new considerations such as the necessity for optimized experiment duration.

Our study has several limitations. As this work is a case study of a real-world implementation, we focused on establishing a functional baseline rather than testing a wide range of parameters. First, we focused on the clinically relevant task of immune phenotyping. It remains to be investigated whether challenges such as the long experiment run time, would also be critical in other CPATH applications, such as Multiple Instance Learning-based slide-level tasks. Second, whereas various FL frameworks exist,^34^, ^35^, ^36^, ^37^ we did not compare these within this study. Third, we did not investigate the impact of changes in experiment parameters such as aggregation method, number of clients, dataset size, as well as parameters relevant for larger federations such as client sampling or asynchronous updates. We stress that running such controlled experiments is often infeasible in an initial real-world clinical study, as the very challenges of extreme experiment duration and complex setup as documented in this article make extensive experimentation prohibitive. We recommend future work to build on our findings to perform controlled studies on the impact of these parameters in a larger-scale CPATH setting. Lastly, we acknowledge that the discussed challenges are non-exhaustive, whereas other aspects such as the development of legal frameworks^33^ remain unaddressed.

In conclusion, our study addresses the pressing need for insights into the practical aspects of utilizing FL for CPATH. By framing our findings as direct consequences of the CPATH-specific constraints, we highlight the real-world challenges of experiment duration, infrastructure design, and experiment management. Future research could expand on this by developing a comprehensive guide for FL in CPATH, covering diverse use cases, software frameworks and institutional settings. This can accelerate the adoption of FL for CPATH applications, ultimately advancing the integration of AI into pathology workflows and improving patient care outcomes.

## Tumor profiler consortium and affiliations

Rudolf Aebersold^5^, Melike Ak^34^, Faisal S Al-Quaddoomi^12,23^, Silvana I Albert^10^, Jonas Albinus^10^, Ilaria Alborelli^30^, Sonali Andani^9,23,32,37^, Per-Olof Attinger^15^, Marina Bacac^22^, Daniel Baumhoer^30^, Beatrice Beck-Schimmer^45^, Niko Beerenwinkel^7,23^, Christian Beisel^7^, Lara Bernasconi^33^, Anne Bertolini^12,23^, Bernd Bodenmiller^11,41^, Ximena Bonilla^9^, Lars Bosshard^12,23^, Byron Calgua^30^, Ruben Casanova^41^, Stéphane Chevrier^41^, Natalia Chicherova^12,23^, Ricardo Coelho^24^, Maya D'Costa^14^, Esther Danenberg^43^, Natalie R Davidson^9^, Monica-Andreea Drăgan^7^, Reinhard Dummer^34^, Stefanie Engler^41^, Martin Erkens^20^, Katja Eschbach^7^, Cinzia Esposito^43^, André Fedier^24^, Pedro F Ferreira^7^, Joanna Ficek-Pascual^1,9,17,23,32^, Anja L Frei^37^, Bruno Frey^19^, Sandra Goetze^10^, Linda Grob^12,23^, Gabriele Gut^43^, Detlef Günther^8^, Pirmin Haeuptle^3^, Viola Heinzelmann-Schwarz^24,29^, Sylvia Herter^22^, Rene Holtackers^43^, Tamara Huesser^22^, Alexander Immer^9,18^, Anja Irmisch^34^, Francis Jacob^24^, Andrea Jacobs^41^, Tim M Jaeger^15^, Katharina Jahn^7^, Alva R James^9,23,32^, Philip M Jermann^30^, André Kahles^9,23,32^, Abdullah Kahraman^23,37^, Viktor H Koelzer^30,37,42^, Werner Kuebler^31^, Jack Kuipers^7,23^, Christian P Kunze^28^, Christian Kurzeder^27^, Kjong-Van Lehmann^2,4,9,16^, Mitchell Levesque^34^, Ulrike Lischetti^24^, Flavio C Lombardo^24^, Sebastian Lugert^14^, Gerd Maass^19^, Markus G Manz^36^, Philipp Markolin^9^, Martin Mehnert^10^, Julien Mena^5^, Julian M Metzler^35^, Nicola Miglino^36,42^, Emanuela S Milani^10^, Holger Moch^37^, Simone Muenst^30^, Riccardo Murri^44^, Charlotte KY Ng^30,40^, Stefan Nicolet^30^, Marta Nowak^37^, Monica Nunez Lopez^24^, Patrick GA Pedrioli^6^, Lucas Pelkmans^43^, Salvatore Piscuoglio^24,30^, Michael Prummer^12,23^, Prélot, Laurie^9,23,32^, Natalie Rimmer^24^, Mathilde Ritter^24^, Christian Rommel^20^, María L Rosano-González^12,23^, Gunnar Rätsch^1,6,9,23,32^, Natascha Santacroce^7^, Jacobo Sarabia del Castillo^43^, Ramona Schlenker^21^, Petra C Schwalie^20^, Severin Schwan^15^, Tobias Schär^7^, Gabriela Senti^33^, Wenguang Shao^10^, Franziska Singer^12,23^, Sujana Sivapatham^41^, Berend Snijder^5,23^, Bettina Sobottka^37^, Vipin T Sreedharan^12,23^, Stefan Stark^9,23,32^, Daniel J Stekhoven^12,23^, Tanmay Tanna^7,9^, Alexandre PA Theocharides^36^, Tinu M Thomas^9,23,32^, Markus Tolnay^30^, Vinko Tosevski^22^, Nora C Toussaint^13^, Mustafa A Tuncel^7,23^, Marina Tusup^34^, Audrey Van Drogen^10^, Marcus Vetter^26^, Tatjana Vlajnic^30^, Sandra Weber^33^, Walter P Weber^25^, Rebekka Wegmann^5^, Michael Weller^39^, Fabian Wendt^10^, Norbert Wey^37^, Andreas Wicki^36,42^, Mattheus HE Wildschut^5,36^, Bernd Wollscheid^10^, Shuqing Yu^12,23^, Johanna Ziegler^34^, Marc Zimmermann^9^, Martin Zoche^37^, Gregor Zuend^38^

^1^AI Center at ETH Zurich, Andreasstrasse 5, 8092 Zurich, Switzerland, ^2^Cancer Research Center Cologne-Essen, University Hospital Cologne, Cologne, Germany, ^3^Cantonal Hospital Baselland, Medical University Clinic, Rheinstrasse 26, 4410 Liestal, Switzerland, ^4^Center for Integrated Oncology Aachen (CIO-A), Aachen, Germany, ^5^ETH Zurich, Department of Biology, Institute of Molecular Systems Biology, Otto-Stern-Weg 3, 8093 Zurich, Switzerland, ^6^ETH Zurich, Department of Biology, Wolfgang-Pauli-Strasse 27, 8093 Zurich, Switzerland, ^7^ETH Zurich, Department of Biosystems Science and Engineering, Mattenstrasse 26, 4058 Basel, Switzerland, ^8^ETH Zurich, Department of Chemistry and Applied Biosciences, Vladimir-Prelog-Weg 1-5/10, 8093 Zurich, Switzerland, ^9^ETH Zurich, Department of Computer Science, Institute of Machine Learning, Universitätstrasse 6, 8092 Zurich, Switzerland, ^10^ETH Zurich, Department of Health Sciences and Technology, Otto-Stern-Weg 3, 8093 Zurich, Switzerland, ^11^ETH Zurich, Institute of Molecular Health Sciences, Otto-Stern-Weg 7, 8093 Zurich, Switzerland, ^12^ETH Zurich, NEXUS Personalized Health Technologies, Wagistrasse 18, 8952 Zurich, Switzerland, ^13^ETH Zurich, Swiss Data Science Center, Wasserwerkstrasse 10, 8092 Zurich, Switzerland, ^14^F. Hoffmann-La Roche Ltd., Grenzacherstrasse 124, 4070 Basel, Switzerland, ^15^F. Hoffmann-La Roche Ltd., Grenzacherstrasse 124, 4070 Basel, Switzerland, ^16^Joint Research Center Computational Biomedicine, University Hospital RWTH Aachen, Aachen, Germany, ^17^Life Science Zurich Graduate School, Biomedicine PhD Program, Winterthurerstrasse 190, 8057 Zurich, Switzerland, ^18^Max Planck ETH Center for Learning Systems, ^19^Roche Diagnostics GmbH, Nonnenwald 2, 82377 Penzberg, Germany, ^20^Roche Pharmaceutical Research and Early Development, Roche Innovation Center Basel, Grenzacherstrasse 124, 4070 Basel, Switzerland, ^21^Roche Pharmaceutical Research and Early Development, Roche Innovation Center Munich, Roche Diagnostics GmbH, Nonnenwald 2, 82377 Penzberg, Germany, ^22^Roche Pharmaceutical Research and Early Development, Roche Innovation Center Zurich, Wagistrasse 10, 8952 Schlieren, Switzerland, ^23^SIB Swiss Institute of Bioinformatics, Lausanne, Switzerland, ^24^University Hospital Basel and University of Basel, Department of Biomedicine, Hebelstrasse 20, 4031 Basel, Switzerland, ^25^University Hospital Basel and University of Basel, Department of Surgery, Brustzentrum, Spitalstrasse 21, 4031 Basel, Switzerland, ^26^University Hospital Basel, Brustzentrum & Tumorzentrum, Petersgraben 4, 4031 Basel, Switzerland, ^27^University Hospital Basel, Brustzentrum, Spitalstrasse 21, 4031 Basel, Switzerland, ^28^University Hospital Basel, Department of Information- and Communication Technology, Spitalstrasse 26, 4031 Basel, Switzerland, ^29^University Hospital Basel, Gynecological Cancer Center, Spitalstrasse 21, 4031 Basel, Switzerland, ^30^University Hospital Basel, Institute of Medical Genetics and Pathology, Schönbeinstrasse 40, 4031 Basel, Switzerland, ^31^University Hospital Basel, Spitalstrasse 21/Petersgraben 4, 4031 Basel, Switzerland, ^32^University Hospital Zurich, Biomedical Informatics, Schmelzbergstrasse 26, 8006 Zurich, Switzerland, ^33^University Hospital Zurich, Clinical Trials Center, Rämistrasse 100, 8091 Zurich, Switzerland, ^34^University Hospital Zurich, Department of Dermatology, Gloriastrasse 31, 8091 Zurich, Switzerland, ^35^University Hospital Zurich, Department of Gynecology, Frauenklinikstrasse 10, 8091 Zurich, Switzerland, ^36^University Hospital Zurich, Department of Medical Oncology and Hematology, Rämistrasse 100, 8091 Zurich, Switzerland, ^37^University Hospital Zurich, Department of Pathology and Molecular Pathology, Schmelzbergstrasse 12, 8091 Zurich, Switzerland, ^38^University Hospital Zurich, Rämistrasse 100, 8091 Zurich, Switzerland, ^39^University Hospital and University of Zurich, Department of Neurology, Frauenklinikstrasse 26, 8091 Zurich, Switzerland, ^40^University of Bern, Department of BioMedical Research, Murtenstrasse 35, 3008 Bern, Switzerland, ^41^University of Zurich, Department of Quantitative Biomedicine, Winterthurerstrasse 190, 8057 Zurich, Switzerland, ^42^University of Zurich, Faculty of Medicine, Zurich, Switzerland, ^43^University of Zurich, Institute of Molecular Life Sciences, Winterthurerstrasse 190, 8057 Zurich, Switzerland, ^44^University of Zurich, Services and Support for Science IT, Winterthurerstrasse 190, 8057 Zurich, Switzerland, ^45^University of Zurich, VP Medicine, Künstlergasse 15, 8001 Zurich, Switzerland.

## CRediT authorship contribution statement

LAS, RBB, VHK, YN, FS, and MN jointly conceived the study, VHK, YN, YK, MR, RV, and HM directed and managed the project. VHK and BS provided immune phenotypes for the TuPro dataset. JR and MZ established the server network infrastructure. LAS, RBB, KW, and FS developed the model training pipeline. LAS, RBB, and AM carried out the federated learning experiments, LAS and RBB evaluated the results. LAS and RBB wrote the manuscript in consultation with MN, YN, VHK, AM, and FS.

## Ethics approval

The Swiss Tumor Profiler is an approved, observational clinical study with BASEC Registration IDs: 2018-02050 (KEK ZH, Switzerland), 2018-02052 (EKNZ, Basel, Switzerland), 2019-01326 (KEK ZH, Switzerland) and 2024-01428 (KEK ZH, Switzerland). The Retrospective cohort is registered in BASEC under IDs 2018-02282 (KEK ZH, Switzerland) and 2024-01428 (KEK ZH, Switzerland). The Dermatology cohort is registered in BASEC under ID 2024-01428 (KEK ZH, Switzerland).

## Code availability

A custom NVFlare component for best model tracking, selection, and early stopping, developed as part of this research, has been contributed and is publicly available via a pull request to the official NVFlare GitHub repository (PR #3506: https://github.com/NVIDIA/NVFlare/pull/3506).

The source code for the underlying cell detection and tumor segmentation algorithms, as well as the trained models, are not publicly available. This is due to proprietary restrictions related to the public–private nature of the collaboration under which this study was performed.

## Declaration of competing interest

This study was financially supported by 10.13039/100004337Roche.

Y.N.: Employee and stock owner of F. Hoffmann-La Roche AG.

F.S.: Employee and stock owner of F. Hoffmann-La Roche AG.

R.B.B.: Employee of F. Hoffmann-La Roche AG.

A.M.: Employee of F. Hoffmann-La Roche AG.

K.W.: Employee of F. Hoffmann-La Roche AG.

J.R.: Employee of F. Hoffmann-La Roche AG.

M.Z.: Employee of F. Hoffmann-La Roche AG.

R.V.: Employee and stock owner of F. Hoffmann-La Roche AG.

Y.K.: Employee of F. Hoffmann-La Roche AG.

M.R.: Employee and stock owner of F. Hoffmann-La Roche AG.

V.H.K.: Principal investigator of the Swiss Digital Pathology Initiative (SDPI); invited speaker for Sharing Progress in Cancer Care (SPCC) and Indica Labs; advisory board of Takeda; sponsored research agreements with Roche and IAG; listed as innovator on patent applications in computational pathology.

L.A.S.: Invited speaker on behalf of Indica Labs.

## Data Availability

This study used three cohorts. Data from the retrospective and dermatology cohort are derived from routine diagnostics and cannot be shared publicly due to institutional data use agreements. Access to these datasets may be considered upon reasonable request and subject to institutional and ethical approvals. IHC whole slide images from the Tumor Profiler cohort will be made publicly available upon publication of the final Tumor Profiler study results at https://eth-nexus.github.io/tu-pro_website/data/.
