# Supplementary material for: Navigating real-world challenges: A case study on federated learning in computational pathology
Source: J Pathol Inform. 2025 Jul 23;18:100464. doi: 10.1016/j.jpi.2025.100464 (PMC12357140; doi:10.1016/j.jpi.2025.100464)
Supplement: Supplementary material [file mmc1.docx]

**Supplementary Materials**

We provide further information with regards to (S1) the datasets used in this study, as well as the data curation protocols, (S2) the task of immune phenotyping and its clinical relevance, (S3) model training, including the exact hyperparameters used and further evaluation metrics.

**S1. Datasets**

In total, we used 169 CD8+ immunohistochemistry (IHC) stained slides derived from three cohorts: (1) the Swiss Tumor Profiler (TuPro) study cohort (n=114, ^1^ and metastatic melanoma cases retrieved from the archives of (2) the Department of Pathology and Molecular Pathology (Retrospective cohort, n=33,^2^), and (3) Department of Dermatology (Dermatology cohort, n=22), University Hospital Zurich. By design, TuPro and Retrospective samples were AP (red chromogen) stained based on the TuPro staining protocol ^2^, at the Department of Pathology and Molecular Pathology, University Hospital Zurich, while Dermatology samples were stained in the research lab of the Dermatology Clinic at USZ with a different protocol for antigen retrieval. Slides were digitized at 40x with the Ventana DP200 scanner (Ventana Medical Systems) for the TuPro and Retrospective cohort and NanoZoomerS360 (Hamamatsu Photonics) for the Dermatology cohort. The differing scanners and staining protocols result in appearance heterogeneity particularly comparing the Dermatology cohort with the other two cohorts (**Supplementary Figure 1**). For each case tumor segmentation and CD8+ cell detection predictions were generated using the HALO^TM^ AI platform (Indica Labs, Albuquerque, NM) as described in ^2^. Immune categories were assessed by expert pathology review (VHK, BS).


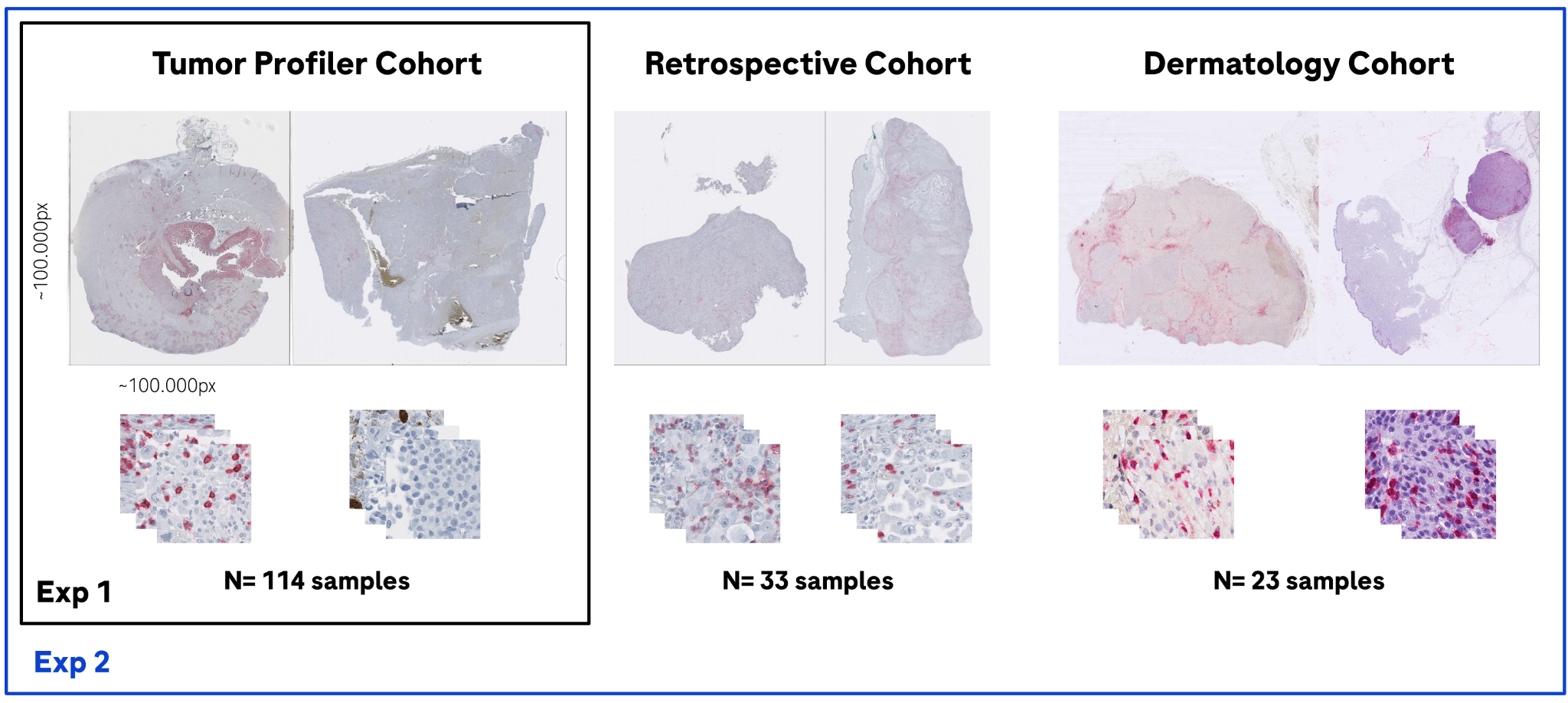


**Supplementary Figure 1:** Cohort appearance: Differences in scanner and staining protocol led to differing appearance of the hematoxylin and AP stain in the three cohorts, shown with a subset of examples

I. Patient-level set definition

Cohorts were split into development and test sets in a 30:70 split on the patient-level, ensuring balanced phenotype distributions (**Supplementary Table 1**). Given the overall limited dataset size and imbalanced class distribution (with <25% inflamed cases), a larger proportion of cases was allocated to the test set to prioritize comprehensive evaluation across phenotypes. Any bias introduced by the smaller training set is monitored and can inform future experimental design. We further split the development set into a patient-level training and validation set with a fixed 70:30 split for **Experiment 1** (**Supplementary Table 2**) and 3 folds for 3-fold cross-validation for Experiment 2 (**Supplementary Table 3**), all with stratified phenotype distributions.

|  | Development Set | | | | Test Set | | | |
| --- | --- | --- | --- | --- | --- | --- | --- | --- |
| Immune Phenotype | Desert | Excluded | Inflamed | Total | Desert | Excluded | Inflamed | Total |
| TuPro (n=114) | 10/34 (29%) | 19/34 (56%) | 5/34 (15%) | **34/114**  **(30%)** | 24/80 (30%) | 43/80 (54%) | 13/80 (16%) | **80/114**  **(70%)** |
| Dermatology (n=22) | 2/9  (22%) | 3/9  (33%) | 4/9  (44%) | **9/22**  **(41%)** | 2/13 (16%) | 4/13  (31%) | 7/13 (53%) | **13/22**  **(59%)** |
| Retrospective (n=33) | 4/13 (31%) | 7/13  (54%) | 2/13 (15%) | **13/33**  **(39%)** | 6/20 (30%) | 11/20 (55%) | 3/30 (15%) | **20/33**  **(61%)** |
| Multi-Cohort (n=169) | 16/56 (28%) | 29/56 (52%) | 11/56 (20%) | **56/169**  **(33%)** | 32/113 (31%) | 48/113 (47%) | 23/113 (22%) | **113/169**  **(67%)** |

**Supplementary Table 1:** Data split of the patient-level development and test datasets and immune phenotype distribution for each cohort. The three cohorts were split into a development set, utilized for training and validation of the deep learning models, and a held-out test set on a patient-level. Cases were split at 30:70 for the TuPro cohort and approximately 40:60 for the Dermatology and Retrospective cohorts. We ensured a similar distribution of the immune phenotypes in each development and test set. The Multi Cohort is the combination of all three cohorts into a single one.

|  | Training Set | | | | Validation Set | | | |
| --- | --- | --- | --- | --- | --- | --- | --- | --- |
| Immune Phenotype | D | E | I | Total | D | E | I | Total |
| Roche Client | 2/7  (29%) | 4/7  (57%) | 1/7  (14%) | **7/24**  **(29%)** | Full validation set | | | |
| USZ Client | 2/9  (22%) | 5/9  (56%) | 2/9  (22%) | **9/24**  **(38%)** | Full validation set | | | |
| LeoMed Client | 3/8  (38%) | 4/8  (50%) | 1/8  (12%) | **8/24**  **(33%)** | Full validation set | | | |
| Full TuPro Set (n=34) | 7/24  (29%) | 13/24  (54%) | 4/24  (17%) | **24/34**  **(71%)** | 3/10  (30%) | 6/10  (60%) | 1/10  (10%) | **10/34**  **(29%)** |

**Supplementary Table 2:** Experiment 1**,** patient-level split of the TuPro development set into training and validation sets for CD8+ cell detection and tumor segmentation. We ensured a similar distribution of the immune phenotypes in each set. D = Desert, E = Excluded, I = Inflamed.

|  | Fold 1 | | | | Fold 2 | | | | Fold 3 | | | |
| --- | --- | --- | --- | --- | --- | --- | --- | --- | --- | --- | --- | --- |
| Immune Phenotype | D | E | I | Total | D | E | I | Total | D | E | I | Total |
| Roche client: TuPro (n=34) | 5/12 (42%) | 6/12 (50%) | 1/12 (8%) | **12/34**  **(36%)** | 3/11 (27%) | 7/11 (64%) | 1/11 (9%) | **11/34**  **(32%)** | 2/11 (18%) | 6/11 (55%) | 3/11 (27%) | **11/34**  **(32%)** |
| USZ client: Dermatology (n=9) | 0/3  (0%) | 1/3 (33%) | 2/3 (66%) | **3/9**  **(33%)** | 1/3 (33%) | 1/3 (33%) | 1/3 (33%) | **3/9**  **(33%)** | 1/3 (33%) | 1/3 (33%) | 1/3 (33%) | **3/9**  **(33%)** |
| LeoMed Client: Retrospective (n=13) | 1/4 (25%) | 2/4 (50%) | 1/4 (25%) | **4/13**  **(31%)** | 1/5 (20%) | 3/5 (60%) | 1/5 (20%) | **5/13**  **(38%)** | 2/4 (50%) | 2/4 (50%) | 0/4 (0%) | **4/13**  **(31%)** |

1. CD8+ cell detection

|  | Fold 1 | | | | Fold 2 | | | | Fold 3 | | | |
| --- | --- | --- | --- | --- | --- | --- | --- | --- | --- | --- | --- | --- |
| Immune Phenotype | D | E | I | Total | D | E | I | Total | D | E | I | Total |
| Roche client: TuPro (n=34) | 3/12 (25%) | 7/12 (58%) | 2/12 (17%) | **12/34**  **(36%)** | 7/11 (64%) | 3/11 (27%) | 1/11 (9%) | **11/34**  **(32%)** | 0/11 (0%) | 9/11 (82%) | 2/11 (18%) | **11/34**  **(32%)** |
| USZ client: Dermatology (n=9) | 0/3  (0%) | 1/3 (33%) | 2/3 (67%) | **3/9**  **(33%)** | 1/3 (33%) | 1/3 (33%) | 1/3 (33%) | **3/9**  **(33%)** | 1/3 (33%) | 1/3 (33%) | 1/3 (33%) | **3/9**  **(33%)** |
| LeoMed Client: Retrospective (n=13) | 1/4 (25%) | 2/4 (50%) | 1/4 (25%) | **4/13**  **(31%)** | 2/4 (50%) | 2/4 (50%) | 0/4 (0%) | **4/13**  **(31%)** | 1/5 (20%) | 3/5 (60%) | 1/5 (20%) | **5/13**  **(38%)** |

1. Tumor segmentation

**Supplementary Table 3:** Experiment 2, patient-level development set split into 3 folds for 3-fold cross validation **a.** CD8+ cell detection, **b.** Tumor segmentation. We ensure a similar distribution of the immune phenotypes in each fold. D = Desert, E = Excluded, I = Inflamed.

II. FOV-level set definition

Due to the gigapixel size of the slides we extract a subset of fields of views (FOVs) from each case in a semi-automated manner for training and by expert-selection (MN) in an informed manner for testing. FOV resolutions for each task and set are detailed in **Supplementary Table** 4. The semi-automated curation aims to select a subset of training and validation FOVs from the slides of each cohort's development set to enable a more balanced representation of classes for tumor segmentation and CD8+ sparse and rich tissue regions for CD8+ cell detection, as well as exclude regions with artifacts (e.g., folds, blur). This is particularly relevant for tumor segmentation with a high class imbalance in the full datasets (**Supplementary Figure 2**). Further, as the ground truth labels for tumor segmentation and CD8+ cell detection are model predictions of the prototype detailed in ^2^ they can contain errors, which we aim to reduce through the curation step.

|  | **Development FOV** | **Test FOV** | **Magnification** |
| --- | --- | --- | --- |
| **Tumor Segmentation** | 512 x 512 pixels | 2048 x 2048 pixels | x40 |
| **CD8+ cell detection** | 256 x 256 pixels | 1024 x 1024 pixels | x20 |

**Supplementary Table 4**: Development and Test FOV specifications. Due to the gigapixel size of a whole slide image tiling into field-of-views (FOV) and FOV-based algorithm training and inference is necessary.

| 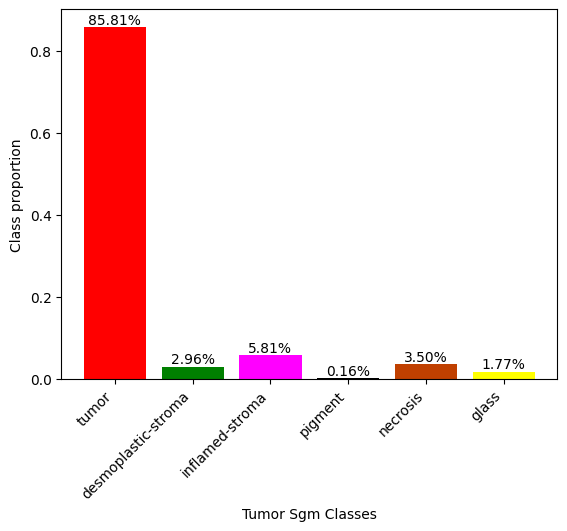   1. Dermatology cohort | 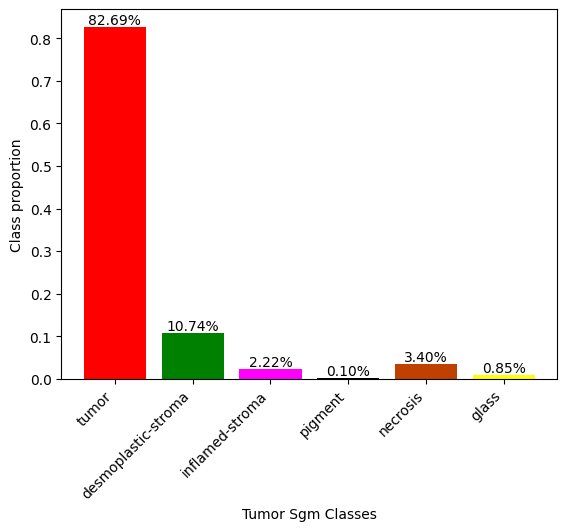   1. Retrospective cohort |
| --- | --- |
| 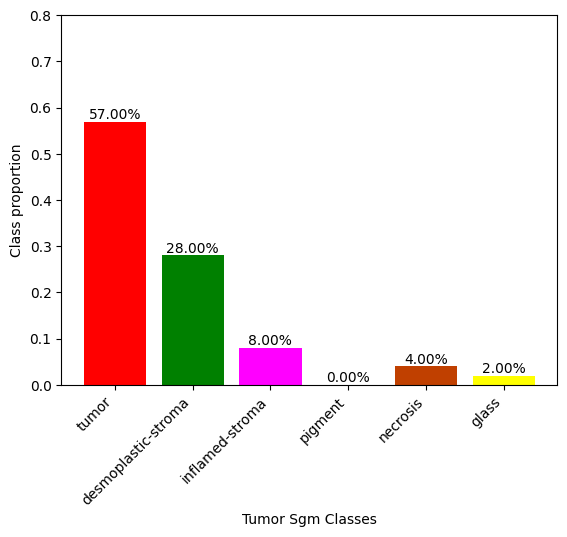   1. TuPro cohort |  |

**Supplementary Figure 2**: Tumor segmentation class distribution in the full development sets.

For the TuPro cohort, FOVs were curated as follows:

- **CD8+ cell detection:**
  - Only use 5% of the total tumor center area of the slide to extract FOVs
  - Only extract FOVs with at least 2 CD8+ cells
  - Random selection of FOVs
- **Tumor segmentation**

For tumor segmentation, a three-step iterative process was applied for data curation, with each step including a visual review:

**Step 1:**

- Only use 5% of the total tumor center area of the slide to extract FOVs
- Random selection of FOVs.
- If the FOV has stroma (either inflamed or desmoplastic), the minimum number of pixels in the FOV for this class is set at >15%
- A percentage of samples in the dataset with a fixed amount of classes was defined (**Supplementary Table 5).**

| **Nº of classes in a FOV** | 1 | 2 | 3 | 4 | 5 | 6 |
| --- | --- | --- | --- | --- | --- | --- |
| **% of samples from the dataset** | 10% | 25% | 30% | 20% | 10% | 5% |

**Supplementary Table 5**: Defined sample percentages based on the amount of classes present in an FOV.

- Dataset lacked adequate representation of blood and necrosis, thus additional FOVs were sampled
  - The minimum class representation for tumor is 10%, desmoplastic stroma is 30% and blood and necrosis is 20%
  - All FOVs must have at least the above defined classes

**Step 2:**

- The Step 1 dataset version was reviewed by a pathologist and image analysis experts and refined as follows:
  - Removing FOVs that comprised quality issues such as regions not in focus or tissue folds
  - Inclusion of more ground truth examples from manually selected larger regions of the WSI to ensure that newly sampled ground truth data would not contain misclassified regions

**Step 3:**

- After visual review and FOV removal in Step 2 the dataset was considerably reduced, particularly the desmoplastic stroma representation had lowered. To prevent re-selection of incorrect ground truth, several ROI were manually annotated by an imaging expert in the WSI on all training slides. The ROIs were selected in an informed manner, ensuring correctness of the ground truth and a higher representation of the desmoplastic stroma class.
- From the ROI regions extract new FOVs with the following criteria:
  - Extract FOVs where at least 1% of this area is pigment
  - Extract FOVs with at least stroma and tumor
    - If stroma is present in the FOV, must be at least 5% of the total area
    - If pigment, glass and blood and necrosis at least 1% of the total FOV area
  - Extract FOVs with desmoplastic stroma, blood and necrosis and tumor
    - If stroma is present in the FOV, must be at least 5% of the total area, blood and necrosis 0.4% and tumor 5%

For the Dermatology and Retrospective cohorts FOVs were selected based on the following criteria:

- **CD8+ cell detection:**
  - Select a maximum 50% of all available FOVs from each case, limited to maximum 300 FOVs per case: $min(300,n_{total FOVs} \cdot50\%)$.
  - Ignore FOVs without CD8+ cells: $w_{FOV}=0$
  - Preference: FOVs with large number of CD8+ cells: $w_{FOV}=\frac{n_{cells in fov}}{n_{cells in wsi}}$
  - FOVs containing folds were ignored upon visual review : $w_{FOV}=0$
- **Tumor Segmentation:**
  - Select a maximum 50% of all available FOVs from each case, limited to maximum 300 FOVs per case: $min(300,n_{total FOVs} \cdot50\%)$.
  - Preference of FOVs with a high fraction of underrepresented classes, to improve class balance:

1. Compute inverse class proportion across whole dataset
   - - - $w_{c}=\frac{N_{total pixels}}{N_{pixels, c}} c \epsilon\{tumor, desmoplastic stroma, inflamed stroma, necrosis, pigment,glass\}$
2. Compute FOV sample weight:
   - - - $w_{underrepresented classes}=\sum_{c} \frac{N_{pixels, c, FOV}}{N_{pixels, FOV}}\cdot w_{c}$
   - Preference of FOVs with balanced classes, i.e., amount of class pixels are balanced. This is based on the observation that small class fractions in FOVs are often an indication of noisy predictions, whereas larger and thereby more balanced class fractions indicate more robust predictions:


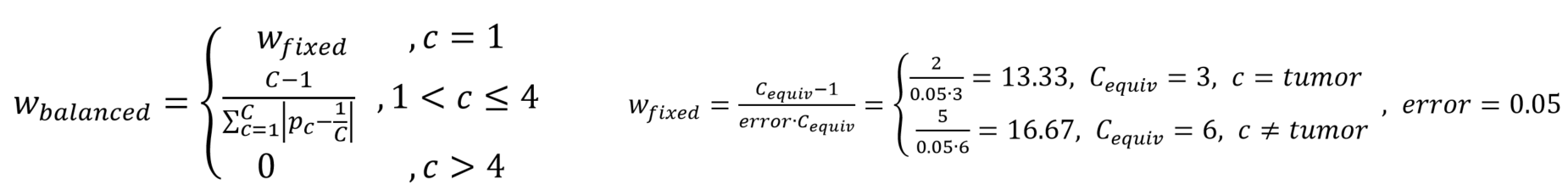


C: num classes present in FOV, $p_{c}$: class proportion for class c

For FOVs with a single class, we assigned a fixed weight, whereas FOVs consisting of only tumor class pixels were assigned a weight equivalent to an FOV with 3 classes all within 0.05 of the ideal proportion; for all other classes we assign a slightly higher fixed weight, equivalent to an FOV with 6 classes within 0.05 of the ideal proportion. This is done to give preference torarer purely non-tumor FOVs over more common purely tumor FOVs. FOVs with more than four classes are ignored, as this is biologically rare and most likely caused by faulty predictions of the research prototype.

- - Combine both weights to the final FOV sample weight:

$$w_{FOV}=\alpha\cdot w_{balanced}+(1-\alpha)\cdot w_{underrepresented}$$

- - We first extracted FOVs with $\alpha=0.5$. Based on a visual review the following additional FOV selection criteria were introduced for tumor segmentation:
    - Ignore FOVs that contain folds
    - Do not sample any desmoplastic stroma from one case due to faulty ground truth.
    - Increase weight for desmoplastic stroma by a factor of 10 for all other cases, $\alpha=0$ to focus on this underrepresented class.

The size of the final curated datasets for per cohort, task and fold are detailed in **Supplementary Table 6**.

|  |  | **Fold 1** | **Fold 2** | **Fold 3** | **Combined** |
| --- | --- | --- | --- | --- | --- |
| **TuPro** | **Tumor Segmentation** | 1751 | 2228 | 2129 | 6108 |
|  | **CD8+ cell detection** | 2416 | 2674 | 2921 | 8011 |
| **Dermatology** | **Tumor Segmentation** | 900 | 900 | 696 | 2496 |
|  | **CD8+ cell detection** | 696 | 871 | 658 | 2225 |
| **Retrospective** | **Tumor Segmentation** | 1200 | 1138 | 1200 | 3538 |
|  | **CD8+ cell detection** | 1180 | 1001 | 997 | 3178 |

**Supplementary Table 6:** Number of FOVs for Tumor Segmentation and CD8+ cell detection for each cross-validation fold.

**S2. Digital Immune Phenotyping**

The CD8+ T lymphocytes infiltration, i.e., their amount and spatial distribution in the tumor microenvironment is debated as a novel predictive biomarker in immune oncology, particularly in melanoma. A favorable response to treatment with immune checkpoint inhibitors (ICI) has been suggested to be more likely for tumors with extensive infiltration (so-called "inflamed" or "hot" tumors). In contrast, tumors with little to no infiltration (referred to as immune excluded or desert, clinically “cold” tumors) correlate with a lack of response to ICI ^3,4^.

The visual evaluation of CD8+ T cells in WSIs by pathologists remains cumbersome and poorly reproducible. However, DL algorithms are being developed to enable automated, reproducible, and objective immune infiltration scoring ^2^. Building on ^2^, we utilize a two-step Digital Immune Phenotyping (DIP) approach based on CD8+ IHC-stained WSIs, consisting of: (1) a tumor segmentation model that identifies tumor, desmoplastic and immune-inflamed stroma, as well as the artifact classes (glass, blood and necrosis, pigment) and (2) a CD8+ cell detection model (**Figure 1**). Combined, this approach provides a spatial estimation of the CD8+ cells densities in the tumor (iCD8+) and in its microenvironment, i.e., in stroma (sCD8+). The resulting densities of iCD8+ and sCD8+ counts are continuous values from which immune phenotypes can be derived.


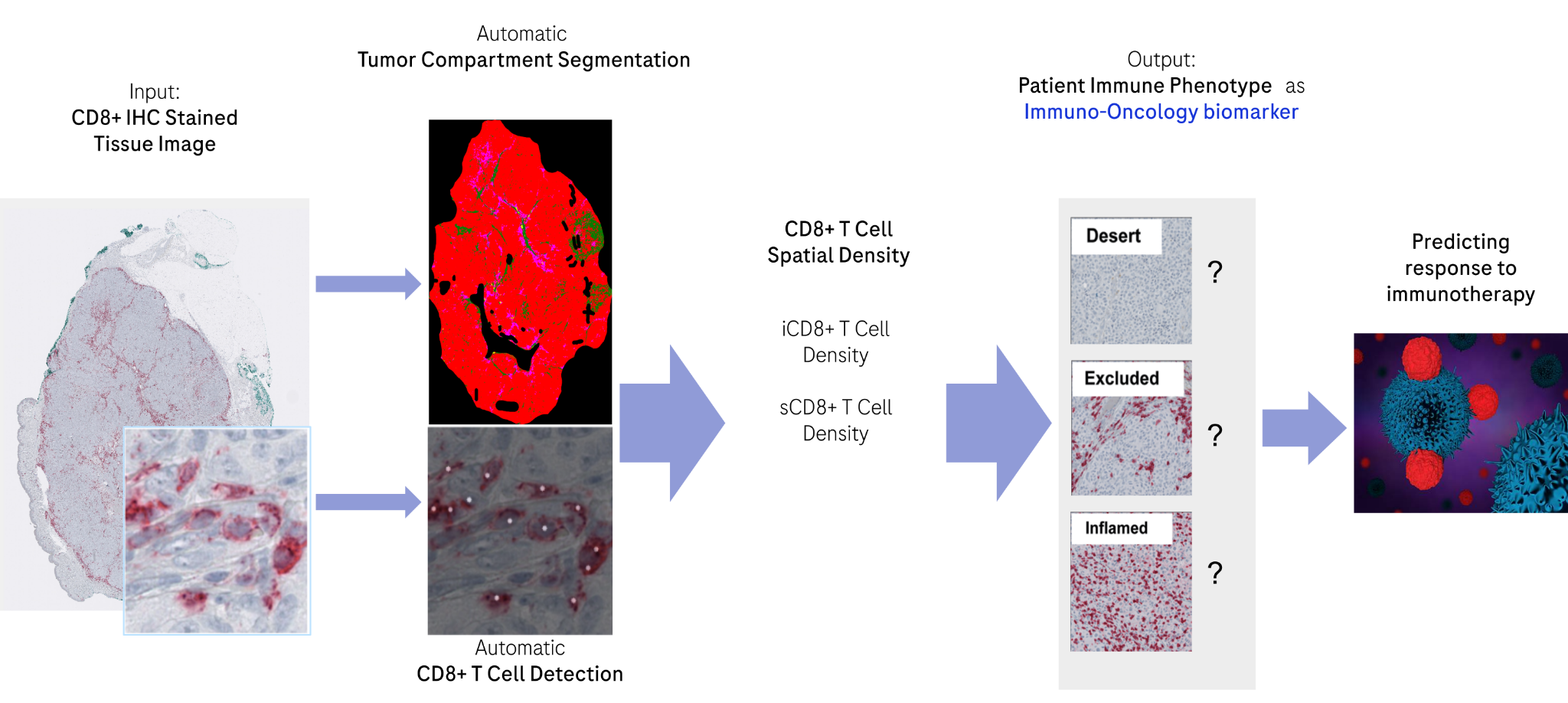


**Supplementary Figure 3:** Automated, DL-based Immune Phenotyping: The tumor core regions are annotated in the CD8+ stained IHC WSIs by pathologists, followed by a DL-based tumor compartment segmentation and CD8+ T cell detection. The results are combined into intratumoral (iCD8+) and stromal (sCD8+) CD8+ T cell densities, from which the patient's immune phenotype can be derived.

**S3. Model training**

DL training was conducted utilizing a U-Net ^5^ based architecture for both tasks (**Supplementary Figures 3**). For cell detection the predicted cell probability map was post-processed into cell centroid coordinates by identifying the local peaks in the prediction using skimage peak_local_max function with a threshold of 0.5 and min_distance of 7. Training hyperparameters were optimized based on validation performance on a fixed 70:30 case-level, label-stratified train-validation split of the TuPro data (**Supplementary Table 2**), when trained in a centralized manner (identified hyperparameter values in **Supplementary Table 7**). Model convergence was monitored based on the F1 score on the validation set(s) (**Table 2**). Early stopping was employed if performance did not improve for N consecutive epochs in centralized or N FL rounds in federated training, N is experiment-dependent and detailed in **Table 1**.


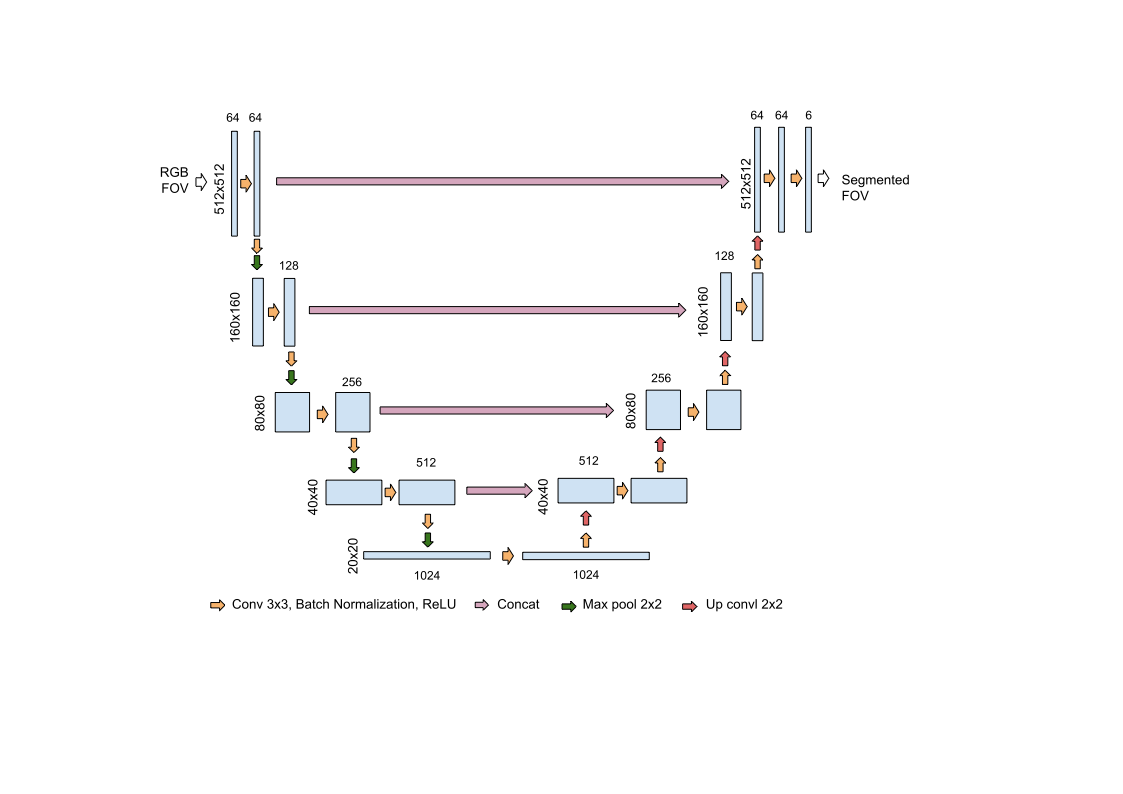


1. U-Net architecture: Tumor segmentation


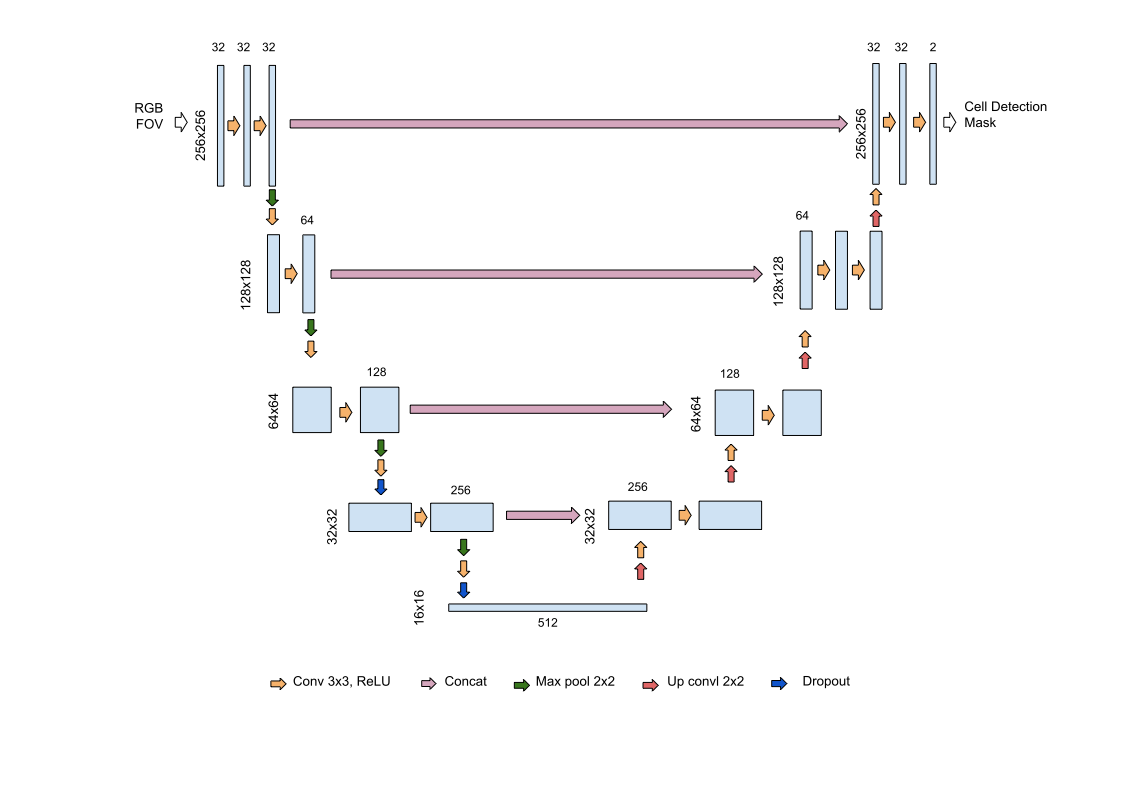


1. U-Net architecture: CD8+ cell detection

**Supplementary Figure 3:** U-Net architectures for (a) tumor segmentation and (b) CD8+ cell detection: Both model architectures for CD8+ cell detection and tumor segmentation were based on a padded U-Net with a depth of 5 layers. For tumor segmentation we define the feature dimension of the first layer to 64, for CD8+ cell detection to 32 and then increase by a factor 2 with increase in depth. We utilized the transpose convolution for the up convolution.

|  | **Optimizer** | **Loss** | **Global Batch Size**  **(at which gradients are updated)** | **Local Batch Size**  **(at which GPU processes samples)** | **LR Scheduler** | **Data Normalization** | **Data Augmentation** |
| --- | --- | --- | --- | --- | --- | --- | --- |
| **Tumor Segmentation** | Adam with weight decay (AdamW)  Learning rate: $10^{-5}$  Weight decay: $5*10^{-8}$  AMSGrad: disabled | Cross entropy loss with class weights* | 24 | Roche: 12  LeoMed 8  USZ:  4 | Triangular mode  Base learning rate $10^{-6}$  Maximum learning rate $10^{-3}$  Step size down 1660  Step size up 1660  Cycle momentum is disabled | ImageNet normalization values, mean = [0.485, 0.456, 0.406] and std = [0.229, 0.224, 0.225] | Image Flip (horizontal, vertical or both), Transpose (swap rows and columns), Random Rotation 90 degrees and Random Brightness and Contrast with limit to 20% |
| **CD8+ cell detection** | Adam  Learning rate: $10^{-4}$ | Cross Entropy Loss | 16 | 16 | Cosine Annealing  Maximum number of iterations 100 | Per R,G, B channel normalization to [0,1] (Division by 255) |  |
| * TuPro: tumor = 0.46021618, desmoplastic stroma = 0.75124627, inflamed stroma = 1.92102463, pigment = 17.40300121, necrosis = 5.52120193 and glass=14.97384069 Retrospective: Inverse, normalized loss weighting: $w_{c, norm}=\frac{w_{c}}{\sum_{c=1}^{C} w_{c}}, w_{c}=\frac{1}{N_{c}}, N_{c}: Number of pixels for class c$  Tumor = 0.002967, desmoplastic stroma = 0.026112, inflamed stroma = 0.05332 pigment = 0.772554, necrosis = 0.057021 and glass=0.088022  Dermatology: Inverse, normalized loss weighting: Tumor = 0.002223, desmoplastic stroma = 0.0171638, inflamed stroma = 0.013652, pigment = 0.897066, necrosis = 0.027066 and glass = 0.0428293 | | | | | | | |

**Supplementary Table 7:** Training hyperparameters for tumor segmentation and CD8+ cell detection in the centralized and federated training setting. Training hyperparameters were initially selected based on the TuPro cohort centralized training with a fixed training and validation split in **Experiment 1**. We then conducted further experiments for each cohort in the multi-cohort setting of **Experiment 2** based on the first cross-validation split (folds 1+2 for training and fold 3 for validation). We found that for all cohorts the hyperparameters chosen for centralized TuPro training also lead to stable training on the new cohorts. However, we did adapt the class weights for tumor segmentation based on the class distribution at each client

|  | **Tumor segmentation** | | | | **Cell detection** | | |
| --- | --- | --- | --- | --- | --- | --- | --- |
|  | **Tumor** | **Desmoplastic Stroma** | **Inflamed Stroma** | **Artifact** | **Precision** | **Recall** | **F1-Score** |
| **Centralized** | 0.968 | 0.730 | 0.775 | 0.792 | 0.722 | 0.804 | 0.768 |
| **Federated** | 0.958 | 0.672 | 0.768 | 0.721 | 0.827 | 0.744 | 0.783 |

**Supplementary Table 8:** Experiment 1, Class-wise F1 Score for tumor segmentation and further CD8+ cell detection metrics at detection threshold 0.5

| 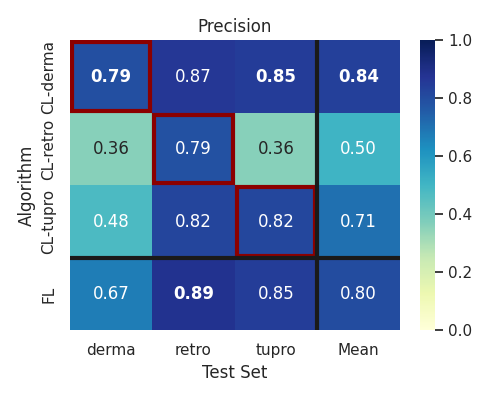 | 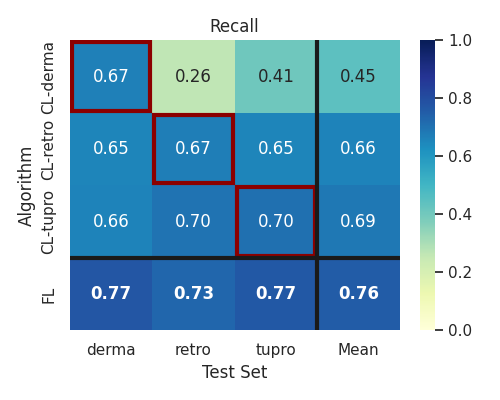 |
| --- | --- |
| 1. Precision | 1. Recall |
| 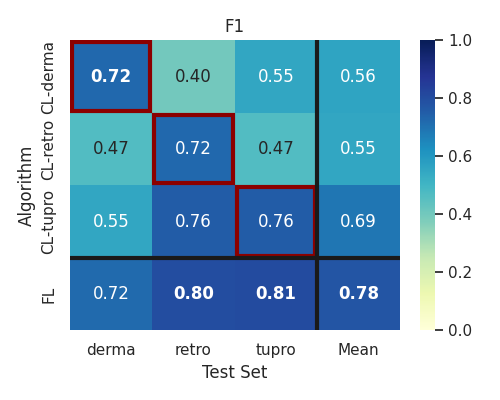 |  |
| 1. F1 |  |

**Supplementary Figure 4:** Experiment 2A, (a) Precision, (b) Recall and (c) F1-Score for CD8+ cell detection at detection threshold 0.5.

| 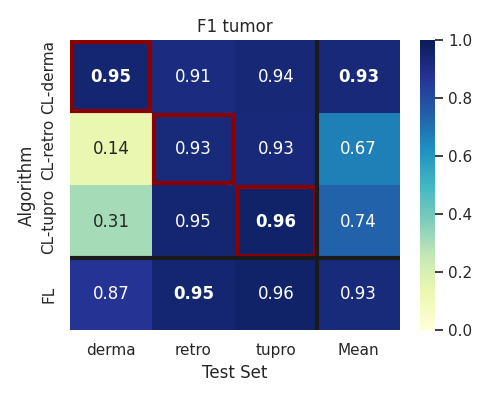 | 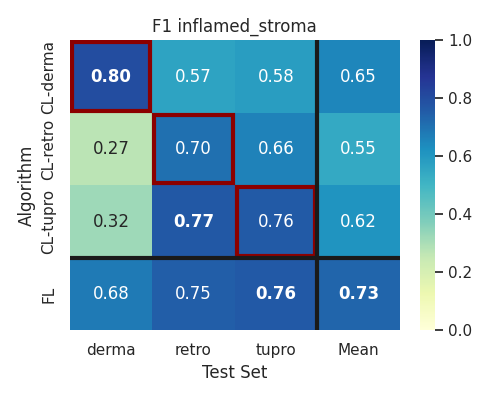 |
| --- | --- |
| 1. Tumor | 1. Inflamed Stroma |
| 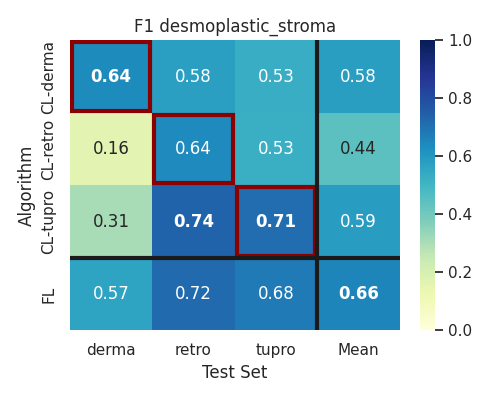 | 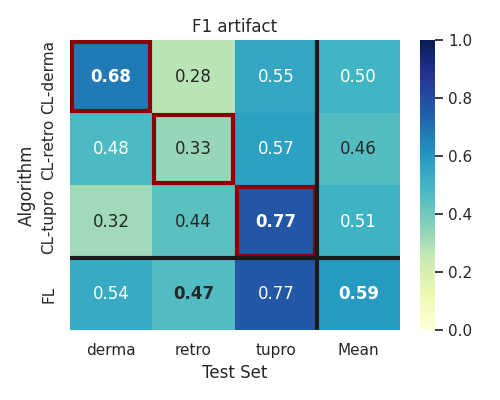 |
| 1. Desmoplastic Stroma | 1. Artifact |

**Supplementary Figure 5:** Experiment 2A, Class-wise F1 Score for tumor segmentation.

| 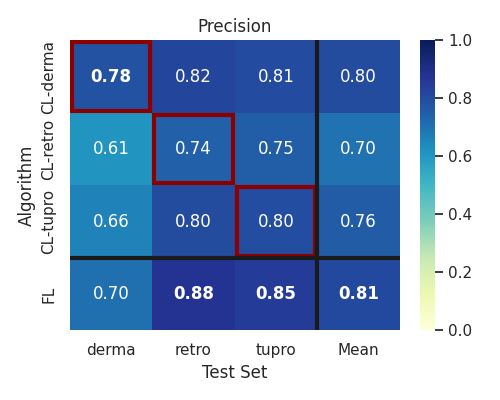 | 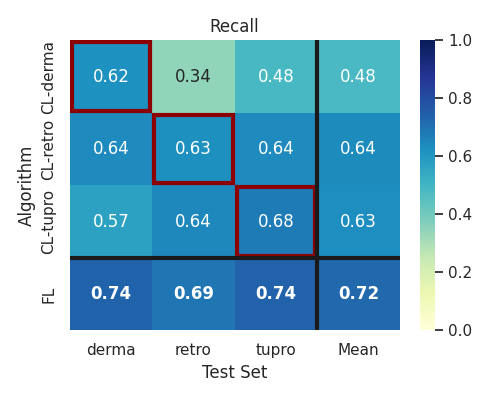 |
| --- | --- |
| 1. Precision | 1. Recall |
| 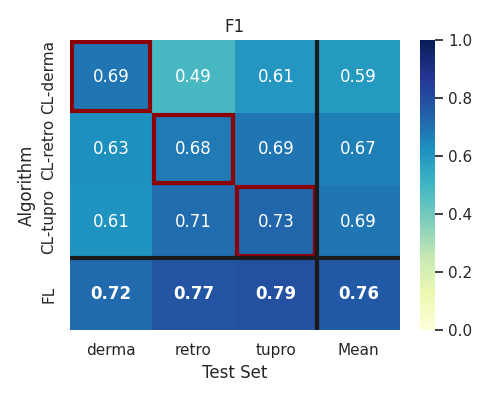 |  |
| 1. F1 |  |

**Supplementary Figure 6:** Experiment 2B, (a) Precision, (b) Recall and (c) F1-Score for CD8+ cell detection at detection threshold 0.5.

| 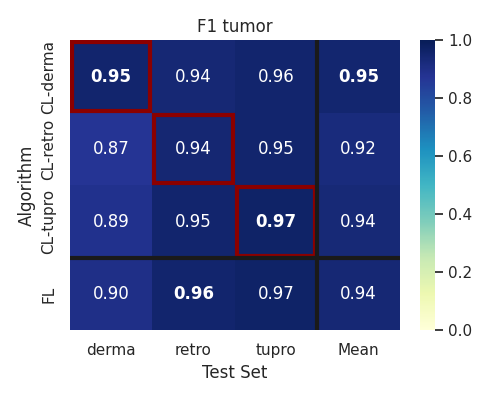 | 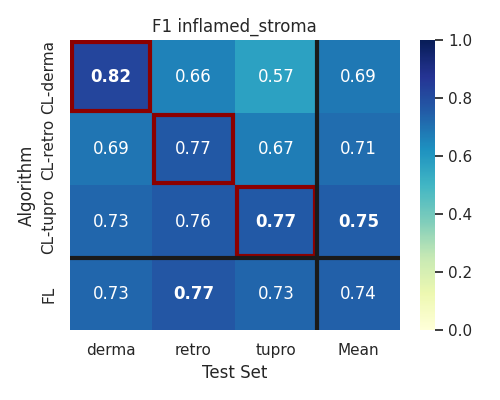 |
| --- | --- |
| 1. Tumor | 1. Inflamed Stroma |
| 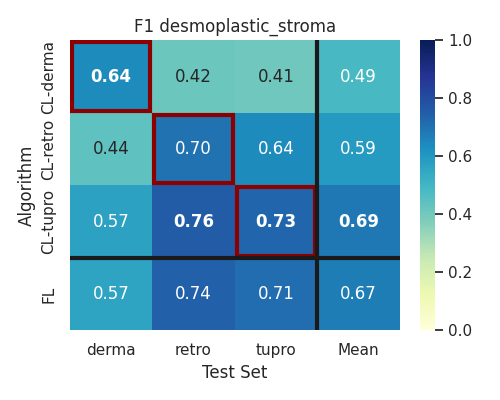 | 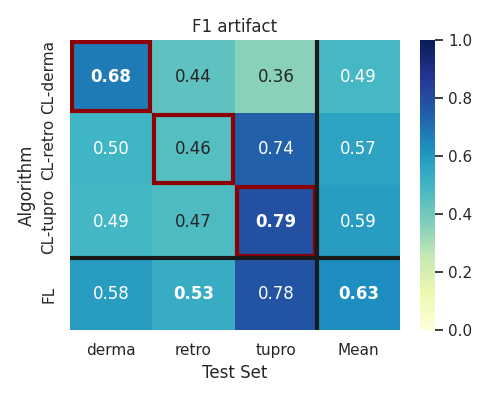 |
| 1. Desmoplastic Stroma | 1. Artifact |

**Supplementary Figure 7:** Experiment 2B, Class-wise F1 Score for tumor segmentation.

|  | Local epochs = 1 | | | | Local epochs = 5 | | | | Local epochs = 20 | | | |
| --- | --- | --- | --- | --- | --- | --- | --- | --- | --- | --- | --- | --- |
| Mean F1 for class | Derma cohort | Retro cohort | TuPro Cohort | Mean | Derma cohort | Retro cohort | TuPro Cohort | Mean | Derma cohort | Retro cohort | TuPro Cohort | Mean |
| Tumor | 0.81 | 0.90 | 0.91 | 0.87 | 0.84 | 0.94 | 0.95 | 0.91 | 0.90 | 0.96 | 0.97 | **0.94** |
| Inflamed stroma | 0.65 | 0.72 | 0.65 | 0.67 | 0.64 | 0.75 | 0.71 | 0.70 | 0.73 | 0.77 | 0.73 | **0.74** |
| Desmoplastic Stroma | 0.15 | 0.36 | 0.35 | 0.29 | 0.44 | 0.64 | 0.59 | 0.55 | 0.57 | 0.74 | 0.71 | **0.67** |
| Artifacts | 0.35 | 0.24 | 0.51 | 0.37 | 0.50 | 0.41 | 0.68 | 0.53 | 0.58 | 0.53 | 0.78 | **0.63** |

|  | Local epochs = 1 | | | | Local epochs = 5 | | | | Local epochs = 20 | | | |
| --- | --- | --- | --- | --- | --- | --- | --- | --- | --- | --- | --- | --- |
| CD8+ cell detection metric  threshold=0.5 | Derma cohort | Retro cohort | TuPro Cohort | Mean | Derma cohort | Retro cohort | TuPro Cohort | Mean | Derma cohort | Retro cohort | TuPro Cohort | Mean |
| Precision | 0.69 | 0.67 | 0.69 | 0.68 | 0.69 | 0.87 | 0.85 | 0.80 | 0.69 | 0.87 | 0.85 | **0.81** |
| Recall | 0.68 | 0.85 | 0.82 | 0.78 | 0.73 | 0.68 | 0.72 | 0.71 | 0.73 | 0.69 | 0.73 | **0.72** |
| F1 | 0.68 | 0.75 | 0.75 | 0.73 | 0.71 | 0.76 | 0.78 | 0.75 | 0.71 | 0.77 | 0.79 | **0.76** |

**Supplementary Table 9:** Experiment 2C, (a) Class-wise tumor segmentation F1 Score and (b) further CD8+ cell detection metrics at detection threshold 0.5, for varying number of local training epochs.

**References**

1. Irmisch A, Bonilla X, Chevrier S, et al. The Tumor Profiler Study: integrated, multi-omic, functional tumor profiling for clinical decision support. *Cancer Cell*. 2021;39(3):288-293. doi:10.1016/j.ccell.2021.01.004

2. Sobottka B, Nowak M, Frei AL, et al. Establishing standardized immune phenotyping of metastatic melanoma by digital pathology. *Laboratory Investigation*. 2021;101(12):1561-1570. doi:10.1038/s41374-021-00653-y

3. Galon J, Bruni D. Approaches to treat immune hot, altered and cold tumours with combination immunotherapies. *Nat Rev Drug Discov*. 2019;18(3):197-218. doi:10.1038/s41573-018-0007-y

4. Wu B, Zhang B, Li B, Wu H, Jiang M. Cold and hot tumors: from molecular mechanisms to targeted therapy. *Sig Transduct Target Ther*. 2024;9(1):274. doi:10.1038/s41392-024-01979-x

5. Ronneberger O, Fischer P, Brox T. U-Net: Convolutional Networks for Biomedical Image Segmentation. Published online May 18, 2015. doi:10.48550/arXiv.1505.04597
